# Diverticular Disease of the Appendix: A Rarely Identified Pathology Prior to Surgery

**DOI:** 10.7759/cureus.93547

**Published:** 2025-09-30

**Authors:** Yannis Karamitas, Jessica Chiang, Alexander H Vu, Sampath Kumar

**Affiliations:** 1 Department of General Surgery, New York University (NYU) Langone Health, Brooklyn, USA

**Keywords:** acute appendicitis, appendectomy, appendiceal diverticulitis, diverticular disease of the appendix, diverticulitis of the appendix

## Abstract

Diverticular disease of the appendix (DDA) remains a rare and poorly understood entity. We present a female patient in her 30s who initially presented for what was believed to be tip appendicitis after imaging. The patient was brought to the operating room (OR) for a laparoscopic appendectomy, and pathology, five days postoperatively, revealed acute appendicitis with DDA. While the patient’s abdominal pain had resolved by this time, her case brings into question how DDA should be managed. DDA holds a greater risk of perforation and has a high association with malignancy. Given its significant association with primary appendiceal malignancy, increased risk of perforation, diverticulitis, and chronic abdominal pain, DDA should be managed with a simple appendectomy when identified intraoperatively. The majority of cases are identified intra- or postoperatively. Future directions include whether a hemicolectomy should be performed if DDA is found. However, failure of early identification remains a current clinical weakness.

## Introduction

Although acute appendicitis is one of the most common surgical pathologies, diverticular disease of the appendix (DDA) remains a rare and poorly understood entity. The original description of DDA can be found as early as 1893 by Kelynack. Case reports written in the 1940s describe the phenomenon, with one author suggesting that surgeons should be suspicious if the appendix is bulbous or club-shaped [1,2]. Presently, there is no consensus on methods for preoperative identification or management of DDA.

Appendicitis is defined as inflammation of the appendix, most often caused by obstruction of the lumen. DDA is classified as either acquired, defined as a “false” diverticulum involving only some layers of the bowel wall, or congenital, a “true” diverticulum involving all layers of the bowel wall. Of the two, the acquired type is more common. Acquired diverticula also tend to be small, 3-5 mm, and appear as single lesions in the distal appendix [3]. DDA has been coined the great imitator, as it mimics the presentation of acute appendicitis, often leading to misdiagnosis. Past studies have cited right lower quadrant (RLQ) pain and nausea as common symptoms of DDA [4,5]. However, a unique finding in DDA is right iliac fossa pain [6-8]. Additionally, unlike the classic appendicitis presentation of diffuse abdominal pain that localizes to the RLQ, DDA pain may not migrate [9]. Patients with DDA further diverge from their acute appendicitis counterparts in that DDA RLQ pain can be more dull and chronic, sometimes manifesting as recurrent attacks of pain [2,10]. In a 2003 report, six patients with chronic abdominal pain underwent extensive workup, including esophagogastroduodenoscopy, imaging, and laboratory studies, without ever achieving a diagnosis for their pain. Following exploratory laparoscopy and appendectomy, all patients had complete resolution of abdominal pain, with histology showing DDA with no signs of inflammation [3]. Appendectomy is the mainstay of treatment in these cases.

The true incidence of DDA is not well quantified. DDA constitutes up to 2.1% of cases of presumed appendicitis, and the general incidence ranges from 0.0014% to 1.9% [1]. However, identifying DDA is important, as it carries a higher risk of both malignancy and perforation. Previous studies have shown that DDA may hold up to a 33% risk of perforation, compared to 10% for acute appendicitis [11]. Overall, malignancy is found histologically in about 0.5%-1% of appendectomy specimens [12]. DDA is associated with mucinous and carcinoid tumors, with one study reporting that 48% of their appendectomy specimens containing DDA harbored a neoplasm [13,14]. DDA occurs more frequently in males than in females and has an inclination for the third to fifth decades of life [3,4]. In addition to age and sex, factors such as past medical history, diet, and family history have also been noted as potentially relevant in its development [3].

The Lipton criterion categorizes DDA into four types: acute diverticulitis, acute appendicitis with acute diverticulitis, acute appendicitis with diverticulum, and appendix with diverticulum. Cases in categories one through three are further subdivided as perforated or non-perforated [15]. Just as the symptoms of DDA mimic acute appendicitis, preoperative imaging often cannot differentiate between the two [16]. Both computed tomography (CT) and ultrasound have been used to detect DDA; however, no studies have been conducted to compare their effectiveness. On CT, DDA has been associated with peri-appendiceal extraluminal air, increased appendix diameter, and greater periappendiceal fat stranding [5]. However, in general, DDA still proves difficult to diagnose preoperatively, as the aforementioned findings can be non-specific and usually very subtle. Given the rarity of DDA and the difficulty in identifying it on imaging, it is often identified histopathologically [2]. The specimen tends to show chronic inflammation and fibrosis, with associated microabscesses, which further confirms that appendiceal diverticulitis tends to have a more prolonged course than acute appendicitis [17]. DDA is associated with primary appendiceal neoplasms, with types ranging from well-differentiated neuroendocrine to low-grade mucinous neoplasms [18].

Although no formal treatment guidelines exist, the literature agrees that prophylactic appendectomy should be performed if DDA is incidentally found [19]. Given reports of primary appendiceal malignancy in up to 48% of patients with DDA, the intraoperative discovery of DDA often raises the question of whether hemicolectomy is warranted. However, the decision to perform a hemicolectomy should not rely on the presence of DDA alone. Instead, it should follow established guidelines for incidentally detected appendiceal malignancy and consider specific factors, such as involvement of the appendiceal base, lymph node status, and tumor histology. Regardless, it is recommended that the entire appendix specimen undergo histopathological examination for further management [19].

This case report has been reported in line with the Surgical Case Report (SCARE) Criteria [20]. This article was previously presented as a meeting abstract at the 2023 Scientific Session of the Society of American Gastrointestinal and Endoscopic Surgeons (SAGES), in Montréal, Canada, between March 19 and April 1, 2023.

## Case presentation

A female patient in the age range of 35-39 years old presented to the Emergency Department with intermittent epigastric and right upper quadrant (RUQ) pain for two weeks. The patient had a past medical and surgical history of active smoking (10-pack years), vertigo, anxiety, laparoscopic cholecystectomy 10 years prior, low transverse C-section nine years prior, and tubal ligation. No family history of diverticular disease was elucidated. Dietary history was unremarkable and non-vegetarian, with no specific restrictions or excessive intake patterns noted. The patient's pain occurred spontaneously, without inciting factors such as food intake. Notably, she did not report RLQ or periumbilical pain.

**Figure 1:**
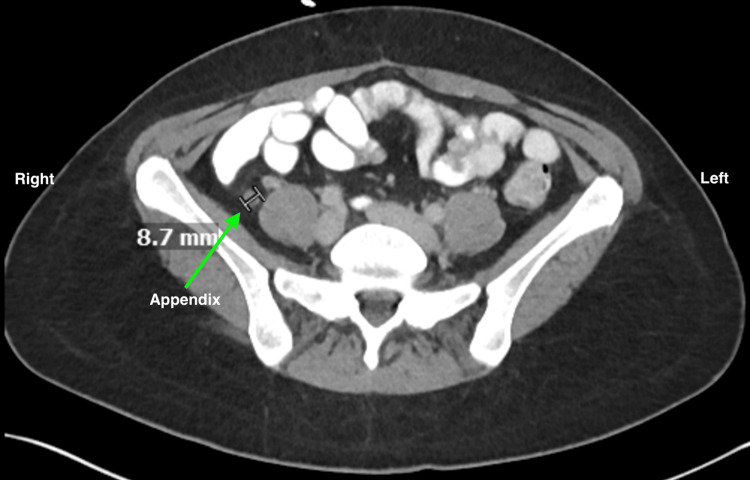
Axial CT scan demonstrating a dilated appendix, with inflammation surrounding the tip, measuring 8.7 mm (arrow). CT, computed tomography

On examination, her vital signs were stable. Laboratory studies showed a white blood cell count of 7.6 × 10³/µL (3.6 × 10³/µL - 11 × 10³/µL), hemoglobin 13.1 g/dL (11.9 g/dL - 16 g/dL), creatinine 0.76 mg/dL (0.51 - 1.15 mg/dL), and normal liver function tests and bilirubin. CT of the abdomen and pelvis with oral and intravenous contrast revealed distal thickening of the appendix measuring up to 0.9 cm, with surrounding inflammatory change concerning for tip appendicitis, as seen in Figure 1. Incidentally, there was a 1-cm hepatic cyst in segment 4A and a calcified granuloma in the spleen.

The patient was admitted to the surgical service and brought to the operating room (OR) for a laparoscopic appendectomy. Entry was achieved using the Hassan technique via a periumbilical blunt port, followed by insufflation and insertion of a 5-mm laparoscope. Dense omental adhesions obscured adequate visualization of the appendix. The patient was placed in steep Trendelenburg, and an additional two 5-mm ports were placed in the left lower quadrant (LLQ) and RUQ. Adhesions were lysed using a LigaSure device (Medtronic, Minneapolis, MN, USA), and an acutely inflamed appendix was encountered. The mesoappendix was serially divided to identify and expose the appendiceal base. The base was then transected using an Endo-GIA stapler (Medtronic, Minneapolis, MN, USA). The specimen was obtained using an endopouch; laparoscopic ports were removed, and the deep fascia at the periumbilical port was closed with #1 absorbable monofilament Maxon. The remainder of the ports were closed, and the patient was discharged on postoperative day 1 in stable condition with no complications. 

**Figure 2:**
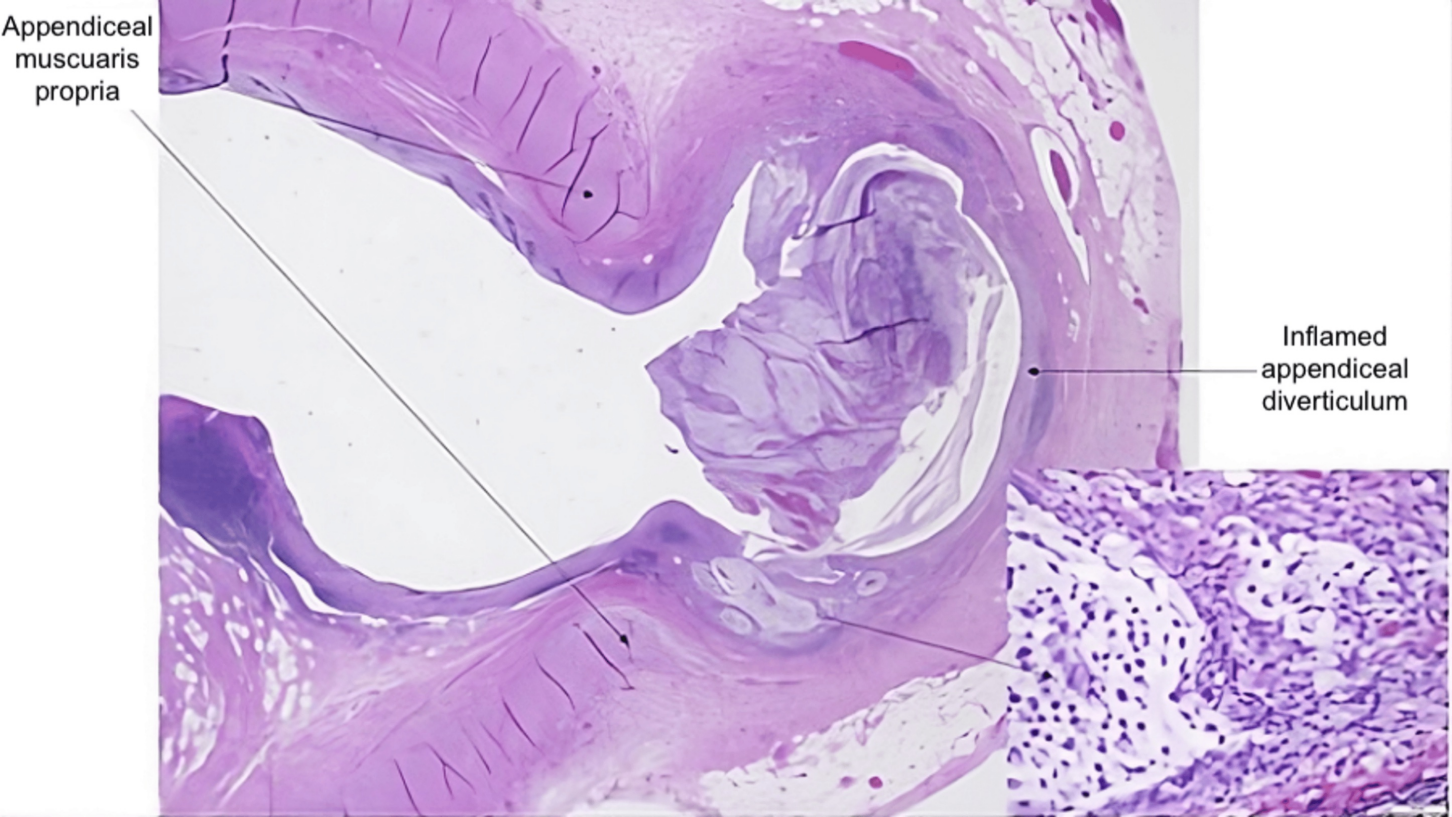
Hematoxylin and eosin-stained sections of the patient’s acquired appendiceal diverticulum, showing mucosal and submucosal herniation through a defect in the muscularis propria (magnifications: 4× and 40×, respectively).

Five days postoperatively, the patient was seen in the outpatient setting for follow-up and discussion of pathology findings. Pathology, as seen in Figure 2, revealed acute appendicitis with DDA and was confirmed after review at a gastrointestinal intradepartmental consensus conference. The specimen measured 6.6 cm in length and 1.1 cm in maximum diameter. Her epigastric and RUQ pain had completely resolved by this time. She tolerated a diet, had normal bowel function, and her incisions were healing well, without signs of infection. At subsequent outpatient follow-up, she remained asymptomatic, with no complications or readmissions, confirming a successful postoperative course. 

## Discussion

The diagnosis in the presented case report can be classified as Lipton 2: acute appendicitis with acute diverticulitis, without perforation. The patient presented with epigastric pain rather than RLQ, or even right iliac fossa, pain, which is usually reported. However, this may be further evidence that DDA is difficult to diagnose preoperatively, as some patients go years without a proper diagnosis [3]. Laparoscopic appendectomy was an appropriate surgery in this case. In a patient with chronic abdominal pain with no definitive diagnosis, even after an extensive work-up, diagnostic laparoscopy can be considered to confirm or exclude DDA. The patient's presentation also prompted consideration of alternative diagnoses, such as peptic ulcer disease, biliary pathology, and gynecologic causes; however, the normal liver function tests, absence of a gallbladder, and unremarkable pelvic findings made these less likely - leaving appendiceal pathology as the most consistent explanation.

The most serious implication of DDA is its association with a wide range of primary appendiceal malignancy subtypes [18]. Therefore, the entire specimen should be analyzed histopathologically to rule out malignancy. The patient fell into the majority, in that her DDA was a postoperative diagnosis. When DDA is recognized preoperatively or intraoperatively, a right hemicolectomy is not warranted if neither imaging nor intraoperative findings raise suspicion for malignancy. In such circumstances, a simple appendectomy is sufficient. If malignancy is identified incidentally on postoperative histopathology, then the decision to proceed with a radical right hemicolectomy should rely on postoperative staging evaluation. Counseling patients about the possibility of DDA-associated malignancy should be considered. Additionally, patient risk factors, such as the risk of loss to follow-up, smoking, and the presence of related comorbidities, should be considered before a more involved surgery is performed. Integrating specific training into surgical residency programs can improve DDA recognition, leading to earlier diagnosis, tailored management, and reduced morbidity and malignancy-related complications.

This case report presents a rare and clinically important pathology which is frequently misdiagnosed as acute appendicitis. Its key strengths lie in discussing the epidemiology, pathophysiology, diagnosis, and management of DDA. The detailed surgical, radiological, and pathological insights regarding laparoscopic setup, imaging, and postoperative histopathologic review by a gastrointestinal intradepartmental conference make this report valuable in showcasing the importance of interdisciplinary collaboration.

Despite the noted strengths, the report has some limitations. One limitation is the lack of a discussion on alternative management strategies, such as surveillance for incidental DDA or non-operative approaches in high-risk surgical patients. Additionally, although the report suggests that hemicolectomy may be appropriate in certain cases, it does not offer a structured treatment algorithm to guide clinical decision-making. Another limitation is the lack of long-term follow-up, as there is no information on the patient’s postoperative surveillance for potential complications.

The consensus for surgical management of DDA is currently appendectomy [13]. Creation of an algorithm to guide management could be a possible next step. Challenges to investigating DDA include its varied presentations and subtle appearance on imaging. Regardless of these characteristics, past reports indicate that DDA should remain on the differential diagnosis in patients with chronic abdominal pain. DDA is a rarely encountered pathology, but its associations with malignancy make it a worthwhile topic for continued research.

## Conclusions

DDA is a rare and often under-recognized condition that can mimic acute appendicitis both clinically and radiographically. This case illustrates that DDA may present with atypical pain patterns, such as isolated epigastric or RUQ discomfort, making preoperative diagnosis particularly challenging. Although imaging raised suspicion for appendicitis, the diagnosis of DDA was only confirmed histopathologically, emphasizing the importance of submitting all appendectomy specimens for thorough analysis. Its markedly higher risk of perforation and notable association with primary appendiceal malignancies highlight the need for careful intraoperative inspection of the appendix and adjacent tissues. When there is no suspicion of malignancy based on imaging or operative findings, a simple appendectomy is sufficient; if malignancy is later identified on histology, further surgical management by right hemicolectomy should follow established oncologic guidelines and staging.

This single case cannot define the true incidence or outcomes of DDA, but it underscores general risks, diagnostic limitations, and the importance of maintaining a high index of suspicion. Greater awareness of DDA among surgeons and pathologists is essential for timely identification and appropriate treatment. Surgeons should consider DDA in the differential diagnosis for patients with recurrent or unexplained abdominal pain, and diagnostic laparoscopy can be a valuable tool when the non-invasive workup is unrevealing. Incorporating DDA into surgical training curricula could improve recognition and reduce complications from delayed diagnosis. Future research should aim to better define its incidence, develop reliable preoperative imaging criteria, and establish evidence-based management algorithms that account for both its benign and malignant potential.
